# Supplementary material for: 2′-Fucosyllactose Ameliorates Oxidative Stress Damage in d-Galactose-Induced Aging Mice by Regulating Gut Microbiota and AMPK/SIRT1/FOXO1 Pathway
Source: Foods. 2022 Jan 7;11(2):151. doi: 10.3390/foods11020151 (PMC8774504; doi:10.3390/foods11020151)
Supplement: Supplementary file 1 [file foods-11-00151-s001.zip › foods-1531632-supplementary.pdf]

foods

Supporting Information 1. Composition of Experimental Diets

Table S1. Composition of experimental diets.

	Control Group	D-gal Group	2'-FL Group
		Ingredient (g/kg Diet)	
Casein	190	190	190
Corn Starch	538	538	535.5
Maltodextrin 10	125	125	125
Cellulose	50	50	50
Corn Oil	50	50	50
Mineral Mix	35	35	35
Vitamin Mix	10	10	10
Choline Bitrartrate	2	2	2
FD&C Yellow Dye	0	0	0.025
FD&C Red Dye	0	0	0.025
2'-Fucosyllactose	0	0	2.5

Supporting Information 2. Real-Time Polymerase Chain Reaction (RT-PCR) Analysis Method

Total RNA was extracted from the tissues (50–100 mg) using Trizol reagent following the manufacturer's protocol. The total RNA was quantified by nanodrop and then reversed to cDNA. Real-time PCR was measured using the SYBR green master mix (Vazyme). The PCR conditions were as follows: (1) heating 95 °C for 30 s; (2) 40cycels at 95 °C for 10 s and 60 °C for 30 s; (3) 60–90 °C for 5 s. Cq was used to calculate the relative expression level of the target gene by the $2^{-\Delta\Delta Cq}$ method. Oligonucleotide primers in RT-PCR were listed in Table S2.

Table S2. Primer Sequences for qPCR.

Gene	Forward	Reverse
β-actin	GTGACGTTGACATCCGTAAAGA	GCCGGACTCATCGTACTCC
TNF-α	CAGGCGGTGCCTATGTCTC	CGATCACCCCGAAGTTCAGTAG
IL-1β	GAAGAAGAGCCCATCCTCTG	GTTCATCTCGGAGCCTGTAG
Claudin-1	AAAGCACCGGGCAGATACAG	CCCAGCAGGATGCCAATTAC
E-cadherin	GGTGAAGGCTTGAGCACAAC	AGGCACTTGACCCTGATACG
MUC2	CTGTGCCAATGGCCTCAAAC	GCCCATCGAAGGTGACAAAG
SIRT1	TAGGGAACCTTTGCCTCATC	TGGCATATTCACCACCTAGC
PGC-1α	CGTTCAAGGTCACCCTACAG	GTTTGGCCCTTTCAGACTCC
FOXO1	TCATCACCAAGGCCATCGAG	TGTTGCTGTCGCCCTTATCC
GPR41	CAAGTTCCAAGCCGACTTTC	TGGATGGCTCTTCTCCATTC
GPR43	GAGGCTGTGGTGTTCAGTTC	GCATAGAGGAGGCAGGATTG
Bcl-2	TCCCGCCTCTTCACCTTTCAG	GTGTTTCCCCGTTGGCATGAG
Bax	CGGCGAATTGGAGATGAACTG	GCAAAGTAGAAGAGGGCAACC
caspase-3	CTCGCTCTGGTACGGATGTG	TCCCATAAATGACCCCTTCATCA
Nrf2	CTTTAGTCAGCGACAGAAGGAC	AGGCATCTTGTTTGGGAATGTG
Keap1	GTGGCACCTACAGAGACACC	GCGCTTGGAGAAGGGCA
HO-1	AGGGCAGAAGGGAATTGCTC	AAAGAGCTGGAGAGCCAACC
NQO-1	GAACCCAGTCTATGCCCCAC	GGCGTGCAAGGGATGATTTC

Supporting Information 3. Western Blot Analysis Method

20 mg of tissue was homogenized and lysed by homogenization medium. The homogenization medium was a mixture of RIPA lysate, protease inhibitor, and phosphatase inhibitor (100:1:1). After the homogenate was centrifuged (4 °C, 10,000× *g*, 5 min), the supernatant was collected and frozen at −80 °C for storage. The protein concentration was determined using the BCA kit (Nanjing Jiancheng Bioengineering Institute, Nanjing, China), based on this normalized concentration. 4× loading buffer (Invitrogen) was used to pretreat the protein, boiled at 95 °C for 10 min. The processed protein sample was separated on 12% SDS-PAGE, and then the band of the target protein was transferred to the nitrocellulose filter (NC) membranes. When the transfer was completed, it was blocked with 5% skimmed milk powder at room temperature for 1 h, and then rinsed repeatedly with tris-buffered saline and Tween 20 (TBST) for 3 times. After washing, the membranes were incubated with primary antibodies for 1 h at room temperature. The following antibodies were used: anti-β-actin (1:1000 dilution, 4970T, CST), anti-SIRT1 (1:1000 dilution, 9475T, CST), anti-FOXO1 (1:1000 dilution, 2880T, CST), anti- Phospho-AMPKα (1:1000 dilution, 2535T, CST), and anti-AMPKα (1:1000 dilution, 5831T, CST). The membranes were incubated with primary antibodies were repeatedly washed with TBST 3 times, and then incubated with horseradish peroxidase (HRP)-conjugated secondary antibodies (1:4000 dilution, 31460, Thermo Fisher) for 1 h at room temperature. After the incubation was completed, the membranes were rinsed again with TBST for 3 times. The resulting membranes were finally developed with a pierce ECL western blotting kit (32106, Thermo Fisher), and then photographed and analyzed under the gel imager.

Supporting Information 4.

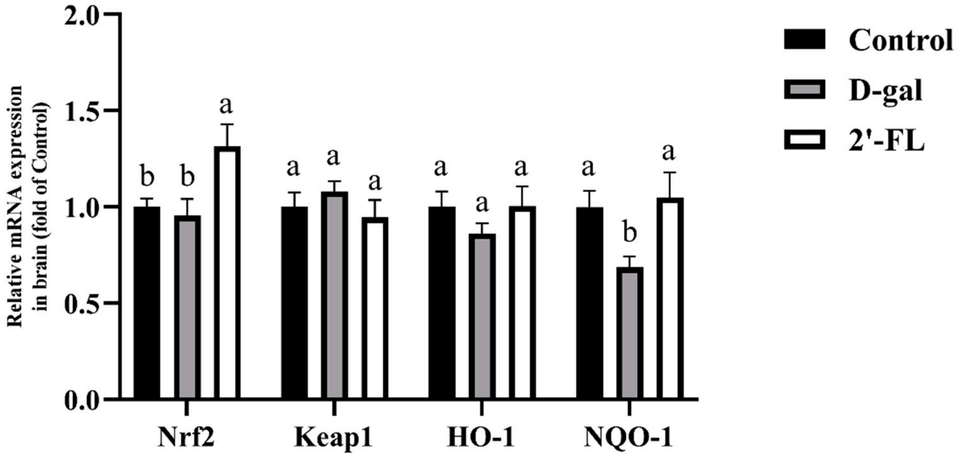

Figure S1. Brain mRNA gene expression of Nrf2 signaling pathway.
